# Evaluation of the theranostic potential of [^64^Cu]CuCl_2_ in glioblastoma spheroids

**DOI:** 10.1186/s13550-024-01084-8

**Published:** 2024-03-07

**Authors:** Catarina I. G. Pinto, André D. M. Branco, Sara Bucar, Alexandra Fonseca, Antero J. Abrunhosa, Cláudia L. da Silva, Joana F. Guerreiro, Filipa Mendes

**Affiliations:** 1 C2TN – Centro de Ciências e Tecnologias Nucleares, Instituto Superior Técnico, Universidade de Lisboa, Lisbon, Portugal; 2 Department of Bioengineering, iBB - Institute for Bioengineering and Biosciences, Associate Laboratory i4HB - Institute for Health and Bioeconomy, Instituto Superior Técnico, Universidade de Lisboa, Lisbon, Portugal; 3 CIBIT/ICNAS Instituto de Ciências Nucleares Aplicadas à Saúde, Universidade de Coimbra, Coimbra, Portugal; 4 ICNAS PHARMA, Universidade de Coimbra, Coimbra, Portugal; 5 Present Address: CIISA - Centro de Investigação Interdisciplinar em Sanidade Animal, Faculdade de Medicina Veterinária, Universidade de Lisboa and Laboratório Associado Para Ciência Animal e Veterinária (AL4AnimalS), Lisbon, Portugal; 6 DECN – Departamento de Engenharia e Ciências Nucleares, Instituto Superior Técnico, Universidade de Lisboa, Lisbon, Portugal

**Keywords:** Copper-64 chloride, Theranostics, Radiobiology, Glioblastoma, Spheroids

## Abstract

**Background:**

Glioblastoma is an extremely aggressive malignant tumor with a very poor prognosis. Due to the increased proliferation rate of glioblastoma, there is the development of hypoxic regions, characterized by an increased concentration of copper (Cu). Considering this, ^64^Cu has attracted attention as a possible theranostic radionuclide for glioblastoma. In particular, [^64^Cu]CuCl_2_ accumulates in glioblastoma, being considered a suitable agent for positron emission tomography. Here, we explore further the theranostic potential of [^64^Cu]CuCl_2_, by studying its therapeutic effects in advanced three-dimensional glioblastoma cellular models. First, we established spheroids from three glioblastoma (T98G, U373, and U87) and a non-tumoral astrocytic cell line. Then, we evaluated the therapeutic responses of spheroids to [^64^Cu]CuCl_2_ exposure by analyzing spheroids' growth, viability, and cells' proliferative capacity. Afterward, we studied possible mechanisms responsible for the therapeutic outcomes, including the uptake of ^64^Cu, the expression levels of a copper transporter (CTR1), the presence of a cancer stem cell population, and the production of reactive oxygen species (ROS).

**Results:**

Results revealed that [^64^Cu]CuCl_2_ is able to significantly reduce spheroids' growth and viability, while also affecting cells' proliferation capacity. The uptake of ^64^Cu, the presence of cancer stem-like cells and the production of ROS were in accordance with the therapeutic response. However, expression levels of CTR1 were not in agreement with uptake levels, revealing that other mechanisms could be involved in the uptake of ^64^Cu.

**Conclusions:**

Overall, our results further support [^64^Cu]CuCl_2_ potential as a theranostic agent for glioblastoma, unveiling potential mechanisms that could be involved in the therapeutic response.

**Supplementary Information:**

The online version contains supplementary material available at 10.1186/s13550-024-01084-8.

## Background

Glioblastoma is the most common primary brain cancer in adults, arising within the central nervous system. It belongs to a heterogeneous family of brain tumors known as gliomas, which derive from glial cells or their precursors [1].

Glioblastoma is classified as a grade IV tumor by the World Health Organization (WHO), being a highly aggressive subtype of glioma with high rates of cell division, abnormal blood vessel growth and central necrotic areas [1]. It is considered incurable, having a very poor median survival of only 14–16 months, even following the combination of several therapeutic strategies including surgical resection, adjuvant radiation therapy (RT) and temozolomide (TMZ) treatment [2].

Additionally, glioblastoma is known to have increased cell proliferation associated with an erratic tumor neovascularization that leads to poor oxygen diffusion, and thus hypoxia [3]. The occurrence of hypoxic regions (< 2% O_2_) leads to the secretion of vascular endothelial growth factor, and, consequently, to angiogenesis, increasing the concentration of several signaling factors in these areas, including copper (Cu) [4]. Indeed, Turecký and colleagues have shown that serum concentrations of Cu were significantly higher in patients with brain tumors than in healthy subjects, being even more increased in patients with more malignant tumors, such as glioblastoma [5].

Copper is a transition metal that is the main building block in more than 30 metalloenzymes and takes part in angiogenesis, a vital process for tumor progression [6]. Furthermore, it alternates between two oxidation states, Cu^+^ and Cu^2+^, generating reactive oxygen species (ROS). When ROS are in excess, these can induce cancer cell cycle arrest, senescence, and apoptosis [4, 7].

Chemically, Cu offers a wide variety of isotopes, such as ^61^Cu, ^62^Cu, ^64^Cu, and ^67^Cu, which can be incorporated in radiopharmaceuticals, being useful for nuclear medicine. Among the isotopes, ^64^Cu has attracted more interest due to its advantageous characteristics [8]. Specifically, ^64^Cu isotope decays to ^64^Zn by β^−^ (electron) emission, or to ^64^Ni by β^+^ (positron) emission or electron capture, which stimulates the emission of Auger electrons [9]. This means that ^64^Cu, a theranostic radioisotope, has the potential to be used for diagnostic purposes, as a Positron Emission Tomography (PET) tracer, and for therapeutic purposes, due to the emission of both β^−^ particles and Auger electrons [10].

^64^Cu labeled diacetyl-2,3-bis(N4-methyl-3-thiosemicarbazone) ([^64^Cu]Cu-ATSM) is a probe that has been successfully used for PET imaging of hypoxic regions of tumors in humans, including glioblastoma [11, 12]. Evidence suggests that high uptake of [^64^Cu]Cu-ATSM correlates with the treatment response and poor prognosis [13]. Additionally, Pérès and colleagues have shown that both [^64^Cu]Cu-ATSM and [^64^Cu]CuCl_2_ accumulate in glioblastoma tumors, not only in hypoxic areas but also in invasive areas closer to the tumor periphery [14]. [^64^Cu]CuCl_2_ is the simplest chemical form of ^64^Cu, being easily available, highly stable, and not requiring complexation with targeting [9, 15].

Both ^64^Cu-based molecules, [^64^Cu]Cu-ATSM and [^64^Cu]CuCl_2_, have been shown to be strongly retained in hypoxic cells of glioblastoma. These are also associated with increased expression of Cu transporters, not only in hypoxic cells but also in non-hypoxic cells [14].

Clinical studies have reported a selective accumulation of [^64^Cu]CuCl_2_ in glioblastoma (within 1 h after intravenous injection) with no adverse or clinically detectable pharmacological effects, supporting the potential of [^64^Cu]CuCl_2_ as a PET imaging agent [16]. Therapeutically, preclinical studies using xenografts of glioblastoma cells implanted in mouse models have shown that administration of a therapeutic dose of [^64^Cu]CuCl_2_ led to a significant tumor decrease (in some cases, complete tumor regression), with a concomitant survival rate increase in treated mice [9].

In the present work, we explored further the potential of [^64^Cu]CuCl_2_ as a theranostic agent for glioblastoma, making use of advanced culture systems, namely multicellular tumor spheroids. Spheroids are a three-dimensional (3D) culture system that can better replicate the in vivo environment of tumor cells. Through the promotion of cell-to-cell and cell-to-extracellular matrix (ECM) interactions, spheroids can re-establish the morphological, functional, and mass-transport properties observed in in vivo tumors [17]. Furthermore, advanced culture models, such as spheroids, are known to replicate the existence of a cancer stem cell (CSC) population that is able to self-renew and differentiate. CSCs are reported to be resistant to conventional chemotherapy and/or radiation therapy, playing an important role in cancer relapse and metastasis formation [15]. The aim of our work was to assess the therapeutic effect of [^64^Cu]CuCl_2_ on growth, viability, and survival capacity of glioblastoma spheroids while trying to elucidate some of the cellular mechanisms involved.

## Methods

### Cell lines and media

Human glioblastoma cell lines T98G and U87 were obtained from the America Type Culture Collection, while U373 was obtained from the European Collection of Authenticated Cell Cultures. Rat Astrocytes (RA) were obtained from Cell Applications, Inc.. T98G and U87 cell lines were cultured in Minimum Essential Medium (MEM) with GlutaMAX™ (Gibco, Thermo Fisher Scientific) supplemented with 10% fetal bovine serum (FBS) (Gibco, Thermo Fisher Scientific). U373 cell line was cultured in the same culture medium further supplemented with 1% non-essential amino acids (Gibco, Thermo Fisher Scientific) and 1% sodium pyruvate (Gibco, Thermo Fisher Scientific). RA cell line was cultured in RA growth medium (Cell Applications, Inc.). All cell lines were grown at 37 °C in a humidified atmosphere of 5% CO_2_ and tested for mycoplasma using the LookOut® mycoplasma Polymerase Chain Reaction (PCR) Detection kit (Sigma-Aldrich, Merk).

### Spheroid culture

Cells were cultured on T25 or T75 culture flasks (Thermo Fisher Scientific). After reaching 80–90% confluence, cell suspensions with the desired cell density were prepared. Two hundred µl of each cell suspension were seeded on Nunclon™ Sphera™ ultra-low attachment 96-well plates (Thermo Fisher Scientific). Cell density per well was 4000, 2000, 1250, and 7000 for T98G, U373, U87, and RA cell lines, respectively. Plates were centrifuged at 405 g for 5 min and incubated at 37 °C in a humidified atmosphere of 5% CO_2_.

### ***[***^***64***^***Cu]CuCl***_***2***_*** solution preparation***

^64^Cu was produced by irradiation of a ^64^Ni target in a medical cyclotron as previously described [18, 19] and supplied as a solution of [^64^Cu]CuCl_2_ in 0.1 M HCl. Prior to the biological studies, the pH of the solution was adjusted to ∼ 7 by adding appropriate volumes of sterile 0.1 M phosphate buffer pH 7.2.

### Spheroids' viability, growth, and circularity determination

Three-day-old spheroids were exposed to 0.56, 1.11, and 2.76 MBq of [^64^Cu]CuCl_2_, by incubation with the [^64^Cu]CuCl_2_ solutions prepared in culture medium and grown for 8 additional days. In the control condition, spheroids were incubated with regular culture medium (0 MBq). Four days after exposure to [^64^Cu]CuCl_2_, half of the exhausted medium was replaced by fresh culture medium, and on the 8th day following exposure, the viability of the spheroids was evaluated using the acid phosphatase (APH) assay, as previously described [15]. Two to four independent assays were performed.

Spheroid growth was monitored over the culture period using a Primovert Inverted ZEISS Microscope, under a total magnification of 40×, with an integrated HDcam camera and using the ZEN 3.5 (blue edition) software. The spheroid area was measured using the software SpheroidSizer [20], and the circularity was calculated according to [1].

1 $${\text{Circularity }} = \, ({4}\uppi \times {\text{Area}})/{\text{Perimeter}}^{{2}}$$

### Colony formation assay

As described above, three-day-old spheroids were exposed to 0.56 and 2.76 MBq of [^64^Cu]CuCl_2_. In the control condition, spheroids were incubated with regular culture medium (0 MBq). After 3 h of exposure, three spheroids per condition were pooled in a microtube, spun down, and the supernatant was discarded. Spheroids were then washed with 500 µl of phosphate saline buffer (PBS) and incubated with 1X TrypLE (Gibco, Thermo Fisher Scientific) at 37 °C for 5 min. Dissociated cells were counted and seeded in 6-well plates (Thermo Fisher Scientific). For T98G and U373 cell lines, 200 cells were seeded for the control conditions and 600 cells for [^64^Cu]CuCl_2_-exposed cells. For the remaining U87 and RA cell lines, 400 cells were seeded for the control conditions and 800 cells for [^64^Cu]CuCl_2_-exposed cells. After an incubation period of 14 days at 37 °C, cells were fixed with a solution of 3:1 methanol:acetic acid (Carlo Erba Reagents and PanReac Quimica CA) at − 20 °C for 20 min. Following washing, fixed cells were stained with 4% Giemsa (Sigma-Aldrich, Merk) in PBS for 10 min. Colonies with more than 50 cells were counted. Results are expressed as the fraction of cellular survival upon treatment compared to the untreated control. Three to five independent assays were performed.

### ***[***^***64***^***Cu]CuCl***_***2***_*** cellular uptake assay***

Cellular uptake studies were performed with three-day-old spheroids. A volume of 100 µl of culture medium was removed from each well, and 100 µl of 0.93 MBq/ml of [^64^Cu]CuCl_2_ in culture medium was added to the spheroids. Spheroids were incubated for 1, 2, and 3 h, at 37 °C. At every time point, 7 spheroids of each cell line were pooled in a microtube, spun down, and the supernatant was removed. Spheroids were then washed with 500 µl of PBS and lysed with 500 µl of 1 M NaOH solution, at 37 °C for 10 min. Radioactivity present in lysed cells was measured using a Hidex AMG Automatic Gamma Counter. The uptake was calculated as the percentage of total activity normalized to spheroid mean area of each cell line and to the uptake of U87 spheroids measured at 1 h. Three to five independent assays were performed.

### Protein extraction and western blot analysis

To determine the basal expression of copper transporter 1 (CTR1), 24 three-day-old spheroids of each cell line were collected and lysed in ice-cold CelLytic™ M Cell Lysis reagent (Sigma-Aldrich, Saint Louis, MO, USA), containing a cocktail of protease inhibitors (Roche Applied Science, Penzberg, Germany). Cellular suspensions were centrifuged at 15,000 g for 15 min and the resultant supernatant was collected. Protein concentration was determined using the DC™ Protein Assay (Biorad, Hercules, CA, USA).

Western blot analysis was performed as previously described [21].

### Immunophenotypic characterization

Immunophenotypic characterization was performed on cells harvested from spheroids. Three-day-old spheroids of each tumor cell line were pooled together and dissociated with Tryple. After washing with PBS, cells were resuspended in FACS buffer (PBS supplemented with 2% FBS) and distributed at a density of approximately 1 × 10^5^ cells/tube. Cells were first incubated with Far Red LIVE/DEAD Fixable Dead Cell Stain Kit (Thermo Fisher Scientific), according to the manufacturer's instructions, to assess cell viability. Following a washing step with FACS buffer, cells were incubated with CD44 PE (clone BJ18, Biolegend) or CD117 PE (clone 104D2, Biolegend) anti-human antibodies, at room temperature for 15 min. After washing, cells were fixed with 100 µl of 2% formaldehyde at 4 °C for 15 min, washed, and resuspended in Fluorescence-Activated Cell Sorting (FACS) buffer. Cells were acquired on a FACSCalibur flow cytometer (BD Biosciences) and data was analyzed using FlowJo v10 software (FlowJo LLC).

### ROS determination

To evaluate the production of ROS after exposure to [^64^Cu]CuCl_2_, three-day-old spheroids were pre-incubated with Fluorobrite™ Dulbecco's modified eagle's medium (DMEM) (Gibco®, Waltham, MA, USA) containing 20 µM 2,7-dichlorodihydrofluorescein diacetate (H_2_DCF-DA) (Invitrogen, Thermo Fisher Scientific) for 1 h at 37 °C. H_2_DCF-DA is a fluorogenic dye (capable of cell membrane permeability) that is converted into 2,7-dichlorodihydrofluorescein (DCF), in the presence of ROS. After incubation, the probe that did not enter cells was discarded and spheroids were then exposed to 0.56, 1.11, and 2.76 MBq of ^64^CuCl_2_. The fluorescent signal of DCF was measured at specific timepoints, using a Varioskan Lux multimode microplate reader (ThermoFisher Scientific, Waltham, MA, USA), at 492 nm excitation and 517 nm emission. The signal of blank wells (with no cells) was subtracted from spheroids' fluorescence signals obtained and the results were expressed as the fold change to the control condition (no exposure to [^64^Cu]CuCl_2_). Two to three independent assays were performed.

### Statistical analysis

GraphPad Prism 9 software was used to perform statistical analysis. Data are shown as mean values ± standard error of the mean (S.E.M.). Statistically significant differences were evaluated considering a threshold of *p*-value = 0.05. One-way analysis of variance (ANOVA), followed by Dunnett's test, was used to determine differences between groups under study.

## Results

### ***Effects of [***^***64***^***Cu]CuCl***_***2***_*** exposure on the growth of glioblastoma spheroids***

Three glioblastoma cell lines were selected to establish spheroids, including two cell lines that are known to be tumorigenic in nude mice, U373 and U87, and a non-tumorigenic line, T98G. RA were used as a non-tumoral control. All the cell lines were able to form compact cell aggregates with a spherical form (Additional file 1: Fig. S1). Spheroids derived from the U87 cell line were the only ones that grew in size throughout the eleven-day culture period. On the other hand, spheroids derived from the other cell lines decreased in size in the initial 4 days and then their mean area stabilized throughout the culture period. On the third day of culture, all spheroids were completely formed, with a compact appearance and a mean diameter ranging from 350 to 450 µm. Considering this, the third day of culture was chosen to start all studies performed in the following sections.

**Fig. 1:**
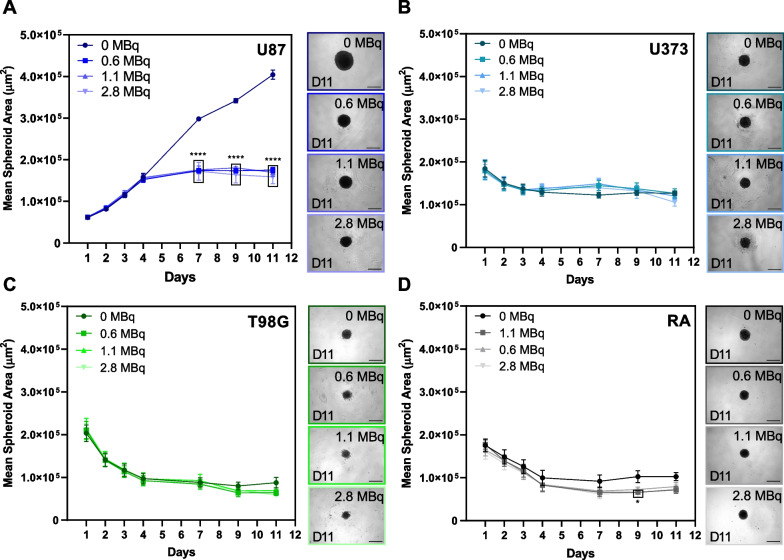
Effects of [^64^Cu]CuCl_2_ exposure on three-day old spheroids.** A–D** Representative microscope images of eleven-day old spheroids exposed to 0, 0.6, 1.1, and 2.8 MBq. Growth curves represented by the mean spheroid area (µm^2^) of U87, U373, T98G, and RA spheroids, respectively, as a function of the number of days in culture are also shown. Scale bar: 500 µm. Data are presented as mean values ± S.E.M. of 2 to 4 independent assays. *p < 0.05, ****p < 0.0001

First, as an initial approach to evaluate the potential therapeutic effect of [^64^Cu]CuCl_2_ on glioblastoma spheroids, we tested if spheroids' growth was affected by exposure to different doses of ^64^Cu. The results obtained revealed that the three cell lines tested had different behaviors after exposure to the radionuclide (Fig. 1). Interestingly, only one of the glioblastoma cell lines—U87—displayed a statistically significant reduction in growth when compared to the non-exposed control (Fig. 1A). In U87 spheroids, growth impairment was independent of the ^64^Cu dose to which spheroids were exposed, being observed after more than 24 h of exposure. On the other hand, spheroids derived from U373 and T98G exhibited no significant changes in their growth behavior when exposed to [^64^Cu]CuCl_2_, in comparison to control conditions (Fig. 1B, C). Nonetheless, the presence of small cellular debris surrounding the spheroids was detectable for all glioblastoma spheroids exposed to the ^64^Cu. This could possibly be the result of partial spheroid disaggregation and cell death, suggesting that ^64^Cu exposure affected spheroids' integrity. In the case of spheroids derived from the non-tumoral cell line (Fig. 1D), exposure to the radioactive compound led to a small decrease in their growth, suggesting that non-tumoral cells can also be damaged by [^64^Cu]CuCl_2_.

To further study the damage caused by ^64^Cu exposure, spheroids' circularity was evaluated (Additional file 1: Fig. S2). While exposure to ^64^Cu led to small changes in their circularity, particularly in the case of U87 and RA spheroids (Additional file 1: Fig. S2A and D, respectively), circularity values remained very close to 1, meaning that all spheroids presented a circular shape throughout time.

### ***Effects of [***^***64***^***Cu]CuCl***_***2***_*** exposure on spheroids' viability and the clonogenic capacity of spheroid-derived cells***

**Fig. 2:**
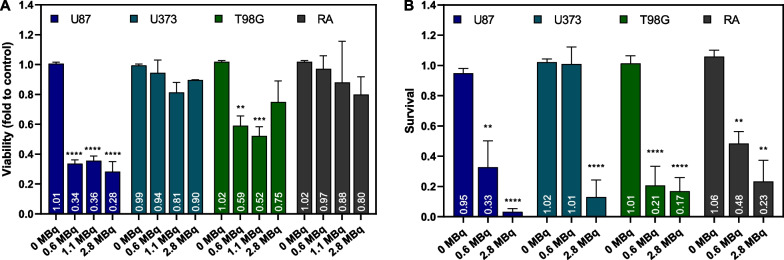
**A** Viability of spheroids derived from U87, U373, T98G, and RA cell lines, evaluated after eight days of [^64^Cu]CuCl_2_ exposure, using the APH assay. Results are presented as the fold change to each cell line's respective untreated control. Data are presented as mean values ± S.E.M. of 2 to 4 independent assays. **B** Survival fraction of spheroid-derived cells from U87, U373, T98G, and RA cell lines after 3 h of [^64^Cu]CuCl_2_ exposure, presented as a fraction of the untreated control, determined through the clonogenic assay. Data are presented as mean values ± S.E.M of 3 to 5 independent assays. **p < 0.01, ***p < 0.001, ****p < 0.0001

To further study the effects of [^64^Cu]CuCl_2_ exposure on glioblastoma spheroids, we evaluated their viability. Spheroid viability was determined eight days after [^64^Cu]CuCl_2_ exposure, using the APH assay (Fig. 2A). In agreement with the abovementioned results, the viability of U87 spheroids was severely affected by exposure to [^64^Cu]CuCl_2_. The decrease in viability (of about 60%) was independent of the dose to which spheroids were exposed. The viability of T98G spheroids was also affected after exposure to [^64^Cu]CuCl_2_, leading to, approximately, a 40% decrease when spheroids were incubated with 0.6 and 1.1 MBq. In the case of U373 and RA spheroids, our results revealed that their viability was less affected by exposure to the radionuclide, suggesting an increased resistance of both cell lines to the treatment.

Assessment of the survival fraction using the clonogenic assay is an established standard assay to determine cell radiosensitivity. Thus, we performed a clonogenic assay to assess the proliferation capacity of cells derived from three-day-old spheroids that were exposed to ^64^Cu for 3 h (Fig. 2B). For cells derived from U87 spheroids, results showed that [^64^Cu]CuCl_2_ exposure affected their proliferative capacity, in comparison to the untreated control condition, demonstrating a high sensitivity towards ^64^Cu exposure. In particular, a correlation was shown between the dose to which spheroids were exposed and the extent to which cell clonogenic capacity was affected. Similarly to U87 cells, T98G cells derived from spheroids exposed to [^64^Cu]CuCl_2_ also had their proliferative capacity severely impaired, in comparison to the untreated control condition. Interestingly, the clonogenic ability of U373 cells was only affected when spheroids were exposed to the highest dose, highlighting an increased resistance to [^64^Cu]CuCl_2_ exposure at lower doses, unlike the other tumor cell lines. In what concerns the non-tumoral cells, RA, it was possible to see that their proliferative capacity was affected by [^64^Cu]CuCl_2_ exposure. Nonetheless, it is important to notice that from all cell lines exposed to the highest dose, RA cells were the ones with the highest survival fraction.

### ***Cellular uptake of [***^***64***^***Cu]CuCl***_***2***_*** in glioblastoma spheroids***

**Fig. 3:**
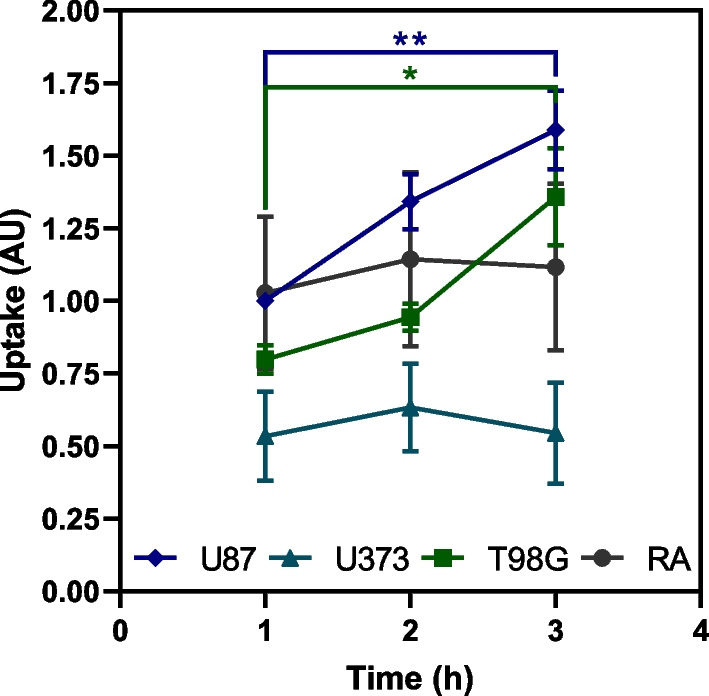
Cellular uptake of [^64^Cu]CuCl_2_ in spheroids derived from glioblastoma and astrocyte cell lines. Cellular uptake was measured at 1, 2, and 3 h after incubation with 0.09 MBq of [^64^Cu]CuCl_2_. Uptake values were normalized to the spheroids' mean area of each cell line and the maximum uptake measured at 1 h in U87 spheroids. Data are presented as mean values ± S.E.M. of 3 to 5 independent assays. *p < 0.05, **p < 0.01

After assessing the possible therapeutic potential of [^64^Cu]CuCl_2,_ we proceeded with mechanistic assays in an attempt to explain the effects observed. As a first approach, studies of cellular uptake were performed with spheroids from all cell lines (Fig. 3), to examine if the damages caused in spheroids could be related with [^64^Cu]CuCl_2_ presence in the cells. Spheroids derived from U87 had the highest uptake, showing increasing values throughout the exposure period. These results further support the abovementioned results of decreased spheroid growth, viability, and proliferative capacity. An identical uptake behavior was seen for T98G spheroids. On the other hand, spheroids derived from U373 had the lowest [^64^Cu]CuCl_2_ uptake throughout the time points. A stable uptake profile was maintained, which can also be associated with the previously observed spheroids' increased resistance. Spheroids derived from the non-tumoral cell line, RA, had the second highest uptake at the two initial time points but maintained a stable uptake profile throughout time.

### Expression of the copper transporter CTR1 in glioblastoma spheroids

**Fig. 4:**
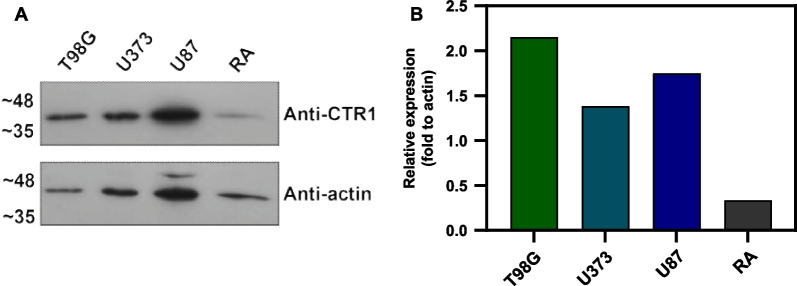
Expression of the copper transporter, CTR1, in T98G, U373, U87 and RA spheroids. **A** Western blot analysis performed to study basal expression levels of CTR1 in spheroids derived from the panel of cell lines selected. Actin was used as a loading control, and detected after stripping of the membrane. **B** Quantification of CTR1 relative expression in comparison to actin expression

We also studied protein expression of one of the transporters responsible for Cu uptake, CTR1, by western blot analysis (Fig. 4 and Additional file 1: Fig. S3). Higher levels of expression of CTR1 were seen in glioblastoma spheroids, in comparison to the non-tumoral cell line, suggesting that ^64^Cu uptake in RA spheroids might be due to alternative mechanisms. Interestingly, among the glioblastoma cell lines, U373 had the lowest expression of CTR1, which might partially explain why spheroids from this cell line had the lowest ^64^Cu uptake under the conditions of our study.

### Expression of cancer stem-like cell markers in glioblastoma spheroids

**Fig. 5:**
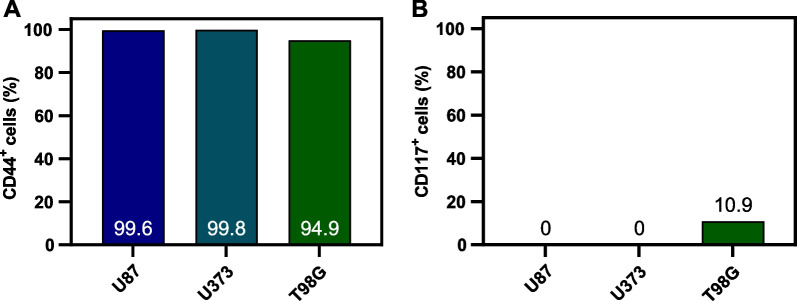
Expression of **A** CD44 and **B** CD117 in three-day old spheroids derived from U87, U373, and T98G cell lines, gated on live cells

To further understand the underlying mechanisms involved in the therapeutic response of glioblastoma spheroids to [^64^Cu]CuCl_2_, we tried to evaluate the possible existence of a CSC population in spheroids. We performed an immunophenotypic characterization, through flow cytometry (Additional file 1: Fig. S4), to evaluate the expression of two surface markers commonly used to characterize the CSC population in glioblastoma, CD44 and CD117 [22, 23]. A clear difference between CD44 and CD117 expression was observed for the three glioblastoma cell lines (Fig. 5). Specifically, nearly all cells in spheroids, derived from every cell line tested, expressed CD44, while expression of CD117 was neglectable in U87 and U373 spheroids. Remarkably, spheroids derived from T98G had a small population of CD117^+^ cells.

### ***Evaluation of ROS production in glioblastoma spheroids exposed to [***^***64***^***Cu]CuCl***_***2***_

**Fig. 6:**
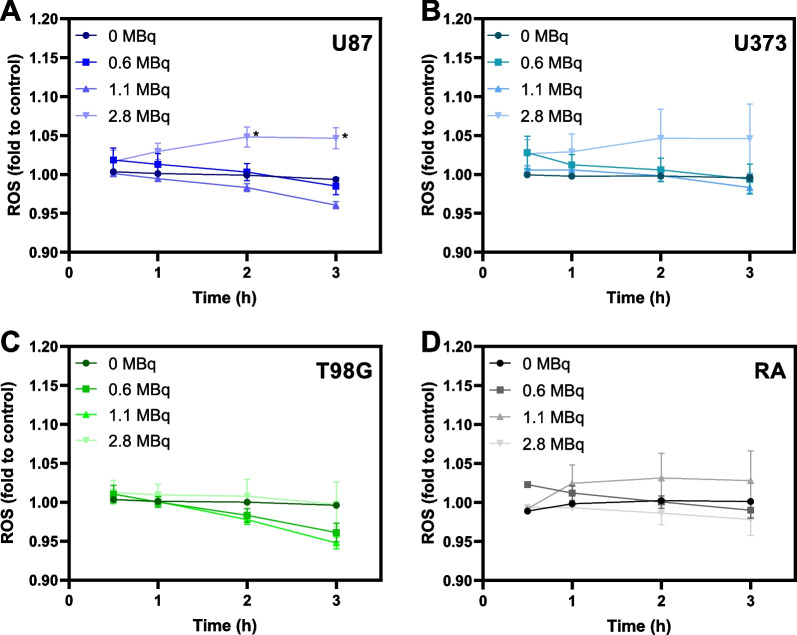
Evaluation of ROS production on three-day old spheroids after exposure to different doses of [^64^Cu]CuCl_2_, probed with H_2_DCFDA. **A–D** ROS production by U87, U373, T98G, and RA spheroids, respectively, using the H_2_DCFDA method based on the detection of DCF fluorescence. Results are presented as the fold change to each cell line's respective untreated control at 1 h as a function of the time of exposure. Data are presented as mean values ± S.E.M. of 2–3 independent assays. *p < 0.05

Ionizing radiation, such as the one resulting from ^64^Cu decay, is known to directly interact with water, generating ROS that can cause cellular damage, ultimately leading to apoptosis. Considering this known effect, we tried to understand if the damage caused in spheroids by [^64^Cu]CuCl_2_ was related to ROS production. The results obtained (Fig. 6) unveiled that exposure to high doses of [^64^Cu]CuCl_2_ triggered increased generation of ROS in spheroids derived from the U87 cell line. Interestingly, ROS production was not significantly increased in the other cell lines exposed to [^64^Cu]CuCl_2_.

## Discussion

The use of [^64^Cu]CuCl_2_ for glioblastoma has been gathering interest among the scientific and medical communities in recent years. Several interesting studies were conducted not only in animal models but also in humans. Most of these studies were focused on the application of this radiopharmaceutical for glioblastoma diagnosis [16, 14]. Nonetheless, despite the recent advances, there are still some pitfalls in the knowledge acquired concerning the mode of action of [^64^Cu]CuCl_2_, mainly when the target application is therapy. To better understand the cellular and molecular mechanisms underlying the action of this compound, we studied the theranostic potential of [^64^Cu]CuCl_2_ for glioblastoma in 3D culture models, namely spheroids. These are a robust alternative to performing cellular studies prior to using animal models, having a much higher predictive value than monolayer cultures, which were once the go-to models for these types of studies [24].

Initially, spheroids were successfully established for all lines selected. These were completely formed by the third day of culture, with a mean diameter ranging from 350 to 450 µm. According to simulations, this size range allows for the establishment of a hypoxic core in spheroids, while avoiding the development of a necrotic core [24, 25]. The presence of a hypoxic core, in which cells are oxygen and nutrient-deprived, is an important characteristic of in vivo tumors that favors angiogenesis. Furthermore, hypoxia (< 2% O_2_) has also been related to enhanced cell invasion capacity, development of metastasis, as well as tumor progression and subsequent increased resistance to treatment [26]. On the other hand, in our case, the presence of a necrotic core (in which cells die due to starvation) is undesirable, considering that it might interfere with [^64^Cu]CuCl_2_ cytotoxic evaluation [27].

We then aimed at assessing if exposure to the radionuclide would affect spheroids, not only in terms of growth but also their viability and proliferation capacity. In parallel, these studies were also conducted in a non-tumoral cell line derived from astrocytes, in an attempt to determine if there was a contrast in the response to ^64^Cu-exposure between tumoral and non-tumoral cells found in the same environment. This is particularly relevant in the case of this compound since the emission of β^−^ particles and Auger Electrons provide an extra radiation burden to the patient [28]. For that comparison, we used readily available cortical astrocytes from neonatal rat origin. While sharing some traits with human astrocytes, rat astrocytes are generally smaller and structurally less complex and diverse. Therefore, direct comparison with the human glioblastoma cell lines under study should be evaluated with caution [29]. Our results revealed that U87 spheroids were the most sensitive to ^64^Cu exposure, namely they presented the lowest viability and cell proliferation capacity and were the only ones having their growth compromised after exposure. On the other hand, spheroids derived from the U373 cell line were the ones that showed the most resistance to radionuclide exposure, only being significantly affected in their proliferative capacity when exposed to the highest dose. In the case of the non-tumoral cell line, even though the spheroids' viability was not severely affected upon ^64^Cu exposure, the clonogenic capacity of spheroid-derived cells decreased, meaning that [^64^Cu]CuCl_2_ might also affect healthy tissues.

In the second phase of this work, we investigated potential mechanisms that could be involved in the response of spheroids to [^64^Cu]CuCl_2_. First, we carried out an uptake study in spheroids derived from all cell lines. The results obtained revealed an increased ^64^Cu uptake in spheroids derived from the most sensitive cell line, while spheroids derived from the most resistant cell line had the lowest uptake. This further supports previous results and suggests that ^64^Cu interaction with cells is related to increased cellular damage. Interestingly, the cell line with the highest uptake was not the one with the highest expression of CTR1, suggesting that there might be other key players involved in ^64^Cu interaction with cells, such as the divalent metal transporter 1 (DMT1) also expressed in glioblastoma cell lines [14, 30, 31]. Even though CTR1 expression was lower in astrocytes, there was a high ^64^Cu uptake by spheroids derived from this cell line. This could be explained by the role astrocytes play as regulators of copper homeostasis in the brain, being described as having increased uptake of Cu^+^, mainly mediated by CTR1 and DTM1 [32].

Next, we tried to understand if the presence of cells with stem-like properties was correlated with spheroids' sensitivity to [^64^Cu]CuCl_2_, considering the role of CSCs in tumors' resistance [33]. In an attempt to identify these populations in glioblastoma spheroids, we selected two markers previously used to identify CSCs in this type of tumor, CD44 and CD117 [34, 35]. CD44 is a transmembrane receptor for glycosaminoglycan hyaluronan, found in healthy tissues but also in glioblastoma [34]. The interaction between CD44 and its ligand is reported to potentially mediate invasion, migration, and chemoresistance in several tumor types [36]. It is known that populations that express this marker are able to generate new tumors similar to the original one, in animal models, while populations that do not express CD44 cannot [34]. Furthermore, Breyer and colleagues have shown that CD44 inhibition affected glioblastoma progression in animal models [37]. In accordance with our results, CD44 expression in U87, U373, and T98G cells was close to 100% in previous studies [37–39], being larger in U373, followed by U87 and T98G cell lines [40]. The other CSC marker selected, CD117, also known as *c-kit*, the receptor tyrosine kinase for stem cell factor (SCF), is involved in neural stem cell survival and stimulation of cell growth in gliomas [41]. To our knowledge, only one study has detected the presence of CD117^+^ cells in one of the three selected glioblastoma cell lines, U87. Specifically, authors detected a very small population in spheroids derived from this cell line (< 1%), similar to our findings [23]. Interestingly, the relative expression of CD117 ligand, SCF, was found to be considerably higher in T98G (measured by quantitative real-time RT-PCR) than in other glioblastoma cell lines [42], which could be related to a larger population of CD117^+^ cells found in this study. As anticipated, cell lines having the highest stemness potential, T98G and U373, were the ones found to be less affected by [^64^Cu]CuCl_2_ exposure. Nonetheless, it is important to notice that CSC identification in tumors is still an evolving field and a consensus has not yet been established concerning which markers should be used to identify this cell population. Other cell surface markers, such as CD133, CD15, integrin α6, L1CAM, and A2B5 have been used to identify CSCs populations in glioblastoma [43]. In the future, a more accurate identification of stem-like cell populations in our glioblastoma spheroids is planned, as well as the identification of possible changes in these populations after [^64^Cu]CuCl_2_ exposure.

An important hallmark of ionizing radiation effects, like the one originated by ^64^Cu decay, is ROS generation. ROS are highly reactive to several cellular macromolecules, such as DNA, being involved in genetic instability induction [44]. Moreover, Cu is a potent oxidant, further contributing to ROS generation [45]. Considering this, and aiming to further understand the damages caused by [^64^Cu]CuCl_2_, we assessed whether exposure to [^64^Cu]CuCl_2_ would lead to ROS generation in glioblastoma spheroids. Unlike what was theoretically expected, [^64^Cu]CuCl_2_ did not drastically increase ROS production in all cell lines. Instead, our results revealed that only exposure to higher doses led to ROS production, being this effect more pronounced in spheroids derived from U87. Bearing in mind that increased intracellular ROS levels lead to apoptosis and senescence [46], the higher ROS generation in U87 spheroids might be one of the key mechanisms involved in the largest decrease of cellular viability and proliferative capacity observed after exposure to [^64^Cu]CuCl_2_.

Importantly, besides the mechanisms here evaluated, there might be other key factors playing a role in the response of glioblastoma spheroids to ^64^Cu. For example, TP53 status might influence tumor cells' resistance to treatment. The TP53 gene encodes a transcription factor (p53) involved in processes such as cellular homeostasis, cell proliferation, and survival, among other functions. When the gene is not mutated, under stress conditions, p53 will promote cell cycle arrest, senescence, and apoptosis, avoiding damaged cell propagation [47]. Interestingly, the most sensitive cell line to ^64^Cu exposure under the conditions of our study, U87, has been described as having a wild-type TP53, while the most resistant cell lines (U373 and T98G) present mutations in this gene [48]. Furthermore, other pathways might also be involved in the decrease of cellular viability and proliferation capacity of glioblastoma cells exposed to [^64^Cu]CuCl_2_, considering that cell lines with a mutant p53 also present sensitivity to the radiopharmaceutical. It has also been reported that the PTEN gene, involved in tumor suppression and regulation of cell growth and survival, is mutated in U373 and T98G, which might also be associated with an increased treatment resistance [49, 50]. In what concerns mutations in DNA repair genes, it is reported that the U87 cell line presents a larger number of single nucleotide variations in genes involved in homologous recombination and non-homologous end joining, important to repair DNA damages caused by radiation, in particular those caused by Auger Electrons [21, 50].

Overall, our results reinforce the potential of [^64^Cu]CuCl_2_ as a theranostic agent for glioblastoma, unveiling an increased therapeutic component that could help improve the prognosis of glioblastoma patients.

## Conclusions

Altogether, our results further support the potential of [^64^Cu]CuCl_2_ as a theranostic agent for glioblastoma. Diagnosis of this type of tumor is possible when lower doses of the radionuclide are administered, while larger doses also cause damage to tumor cells. Nonetheless, there are still some questions that need to be further clarified. For instance, the impact of potential deleterious effects on non-tumoral tissues surrounding the tumors should be evaluated. Novel cellular models with increased robustness concerning the simulation of the brain/tumor environment are under development. These will allow to better investigate the glioblastoma biology inserted in a primitive human brain environment and would majorly increase the value of this type of research [51]. Additionally, it would be important to analyze ^64^Cu distribution within the spheroids and, most relevantly, within the cells, as the range of Auger Electrons is very short and proximity to the nucleus is crucial to achieve a high therapeutic efficacy. Acknowledging these pitfalls, we plan on further developing new studies to elucidate ^64^Cu distribution in spheroids, and also in more complex models that include co-culture with non-tumoral human brain cells. Afterward, animal studies should be conducted to better elucidate the translational potential of this simple radiopharmaceutical.

To our knowledge, this is the first study to explore the radiobiological effects of [^64^Cu]CuCl_2_ exposure in glioblastoma spheroids while also exploring the mechanisms responsible for the therapeutic responses observed. With this study, we reinforced the promising potential of ^64^Cu as a theranostic agent with evidence that demonstrates its therapeutic purpose in advanced culture models with a high predictive value. Overall, the present study provides important insights toward a better understanding concerning the therapeutic effect and mode of action of ^64^Cu in the context of glioblastoma.

### Supplementary Information

**Additional file 1.** Additional figures and analyses.

## Data Availability

Data sharing is not applicable to this article as no datasets were generated or analyzed during the current study.

## Abbreviations

3D: Three-dimensional
64Cu-ATSM: Copper-64 labeled diacetyl-2,3-bis(N4-methyl-3-thiosemicarbazone)
ANOVA: One-way analysis of variance
APH: Acid phosphatase
CSC: Cancer stem cell
CTR1: Copper transporter 1
DCF: 2,7-Dichlorodihydrofluorescein
DMEM: Dulbecco's modified eagle's medium
DMT1: Divalent metal transporter 1
ECM: Extracellular matrix
FACS: Fluorescence-activated cell sorting
FBS: Fetal bovine serum
H2DCF-DA: 2,7-Dichlorodihydrofluorescein diacetate
HRP: Horseradish peroxidase
MEM: Minimum essential medium
PBS: Phosphate saline buffer
PCR: Polymerase chain reaction
PET: Positron emission tomography
RA: Rat astrocytes
ROS: Reactive oxygen species
RT: Radiation therapy
RT-PCR: Real time-polymerase chain reaction
S.E.M.: Standard error of the mean
TMZ: Temozolomide
WHO: World health organization
