# Supplementary material for: Evaluation of the theranostic potential of [64Cu]CuCl2 in glioblastoma spheroids
Source: EJNMMI Res. 2024 Mar 7;14:26. doi: 10.1186/s13550-024-01084-8 (PMC10920519; doi:10.1186/s13550-024-01084-8)
Supplement: Supplementary file 1 — Additional file 1. Additional figures and analyses. [file 13550_2024_1084_MOESM1_ESM.docx]

**Evaluation of the theranostic potential of [^64^Cu]CuCl_2_ in glioblastoma spheroids**

Catarina I. G. Pinto^1^, André D. M. Branco^2^, Sara Bucar^2^, Alexandra Fonseca^3^, Antero J. Abrunhosa^3^, Cláudia L. da Silva^2^, Joana F. Guerreiro^1,4^, Filipa Mendes^1,5^

^1^ C^2^TN – Centro de Ciências e Tecnologias Nucleares, Instituto Superior Técnico, Universidade de Lisboa, Lisbon, Portugal

^2^ Department of Bioengineering, iBB - Institute for Bioengineering and Biosciences - Associate Laboratory i4HB - Institute for Health and Bioeconomy, Instituto Superior Técnico, Universidade de Lisboa, Lisbon, Portugal

^3^ CIBIT/ICNAS Instituto de Ciências Nucleares Aplicadas à Saúde, Universidade de Coimbra, Coimbra, Portugal

^4^ Present address: CIISA - Centro de Investigação Interdisciplinar em Sanidade Animal, Faculdade de Medicina Veterinária, Universidade de Lisboa and Laboratório Associado para Ciência Animal e Veterinária (AL4AnimalS), Lisbon, Portugal

^5^ DECN – Departamento de Engenharia e Ciências Nucleares, Instituto Superior Técnico, Universidade de Lisboa, Lisbon, Portugal

Corresponding author: Filipa Mendes^1,5^ - [fmendes@ctn.tecnico.ulisboa.pt](mailto:fmendes@ctn.tecnico.ulisboa.pt)

**Supplementary Data**


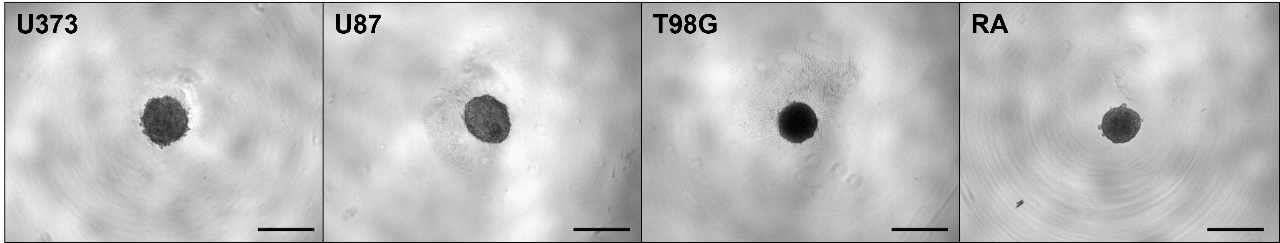


Supplementary Figure S1. Representative microscope images of U373, U87, T98G and RA spheroids at the third day of culture. Scale bar: 500 μm.


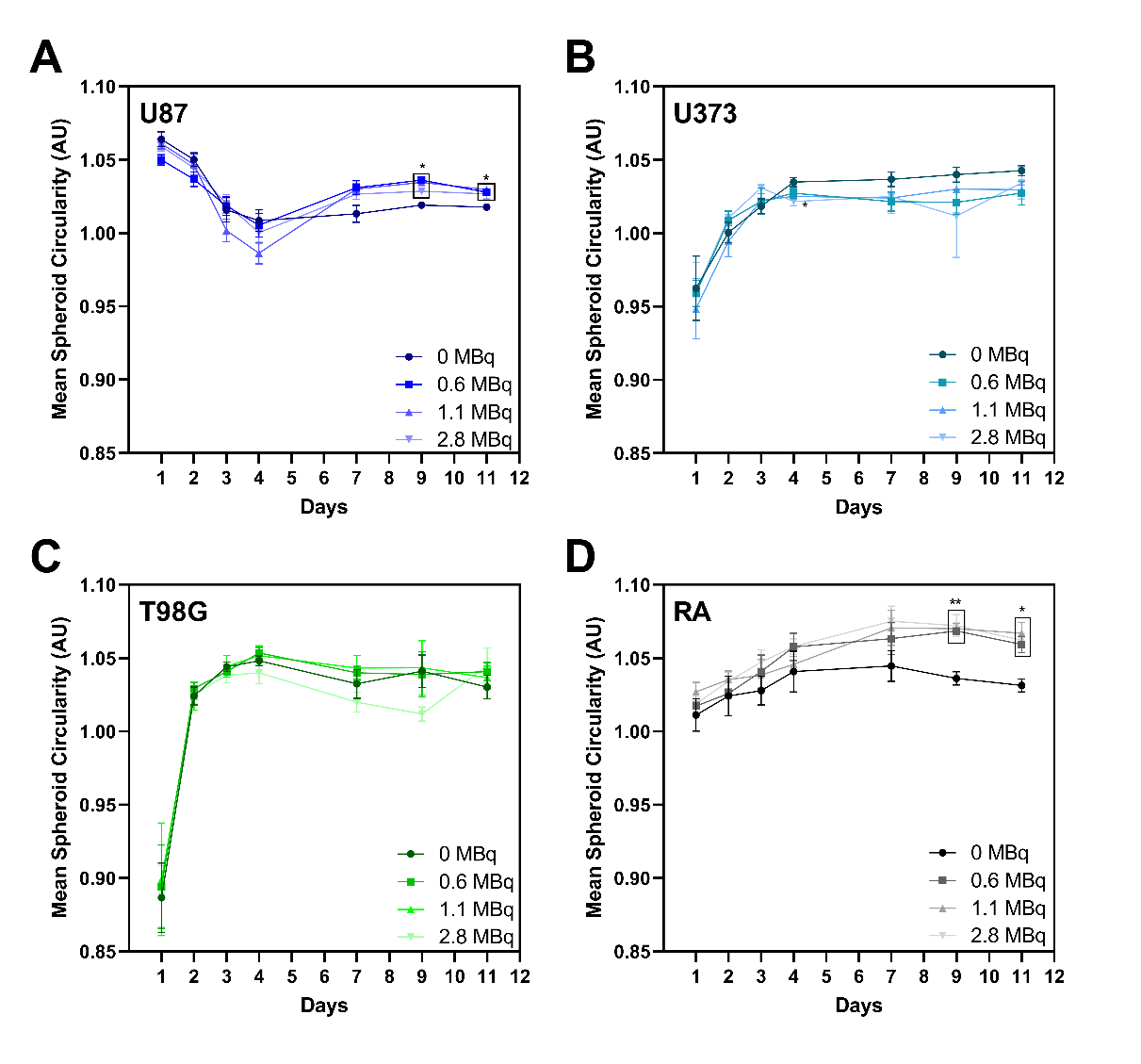


Supplementary Figure S2. Effects of ^64^CuCl_2_ exposure on three-day old spheroids. **(A-D)** Mean spheroid circularity of U87, U373, T98G and RA spheroids, respectively, represented as a function of the number of days in culture. Data are presented as mean values ± S.E.M. of 2 to 4 independent assays. * p < 0.05, ** p < 0.01.


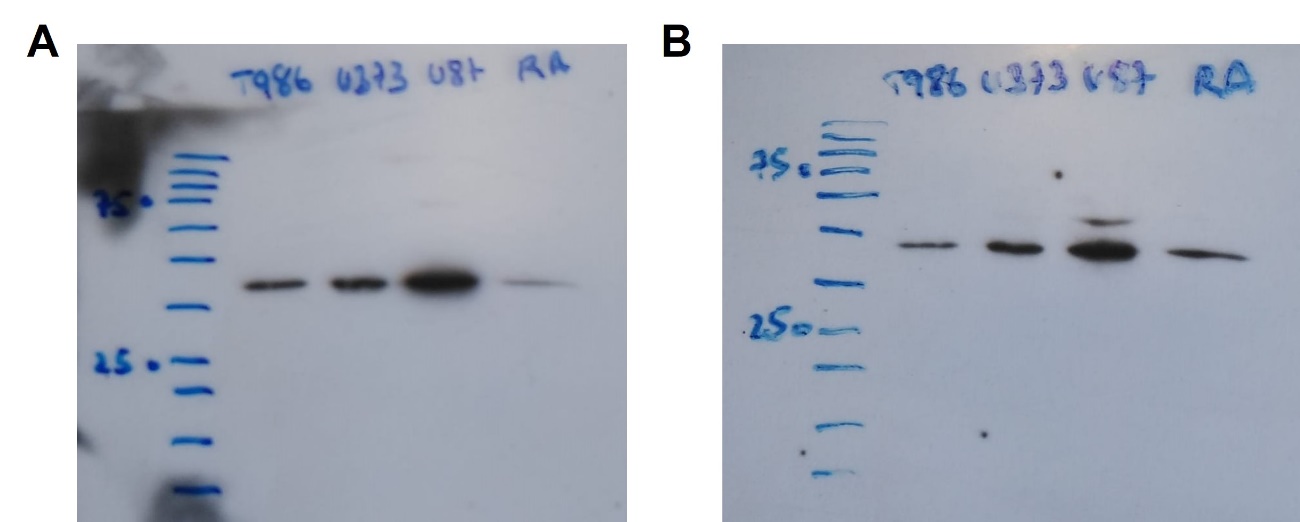


Supplementary Figure S3: Full length Western Blots of the copper transporter, CTR1, and the corresponding loading control, actin, in T98G, U373, U87 and RA spheroids. **(A)** Western Blot CTR1 signal was detected by enhanced chemiluminescence using Pierce^TM^ ECL Western Blotting Substrate. After stripping of the membrane the actin signal **(B)** was detected using the same method.


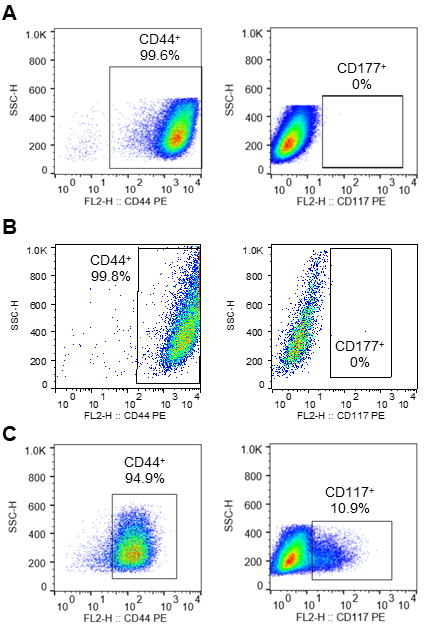


Supplementary Figure S4. Expression of CD44 and CD117 in cells derived from **(A-C)** U87, U373 and T98G spheroids, respectively. SSC – side scatter.
